# Supplementary material for: SARS-CoV-2 Spike and Nucleocapsid Antibody Response in Vaccinated Croatian Healthcare Workers and Infected Hospitalized Patients: A Single Center Cohort Study
Source: Viruses. 2022 Sep 4;14(9):1966. doi: 10.3390/v14091966 (PMC9503044; doi:10.3390/v14091966)
Supplement: Supplementary file 1 [file viruses-14-01966-s001.zip › viruses-1888146-supplementary.pdf]

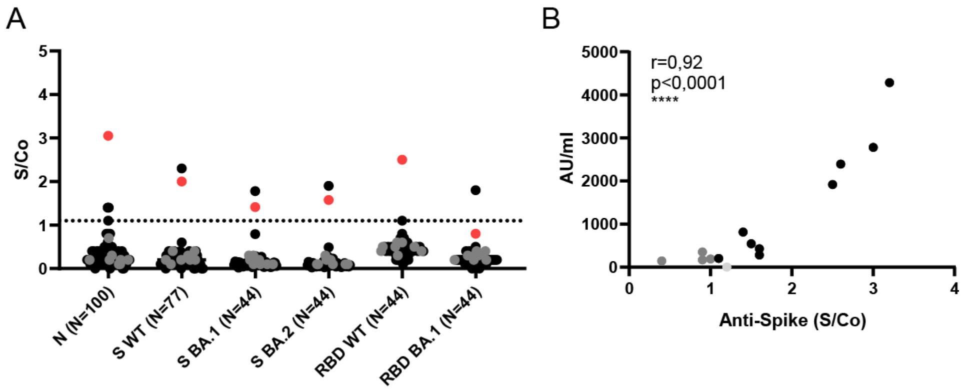

Supplementary Figure S1. (**A**) Determination of cut-off values for Spike and Nucleocapsid ELISAs. Reactivity of IgG in pre-pandemic sera to Nucleocapsid protein (N), full-length Spike protein of WT virus (S), Omicron BA.1 Spike protein (S BA.1) and Omicron BA.2 Spike protein (S BA.2) or Spike RBD protein of WT virus (RBD WT) and Omicron BA.1.1.529 (RBD BA.1). Pre-pandemic sera (black symbols), validated negative controls (grey symbols) and reference positive control serum pool (red symbols). (**B**) Correlation of level of anti-Spike antibodies determined by our full-length Spike ELISA and anti-RBD antibodies determined by reference method (SARS-CoV-2 IgG II Quant, Abbot) for positive samples. S/Co, Signal to cut-off ratio; dashed line indicates cut-off value (S/Co=1,1). Statistical test: Pearson correlation (B), **** p<0.0001.

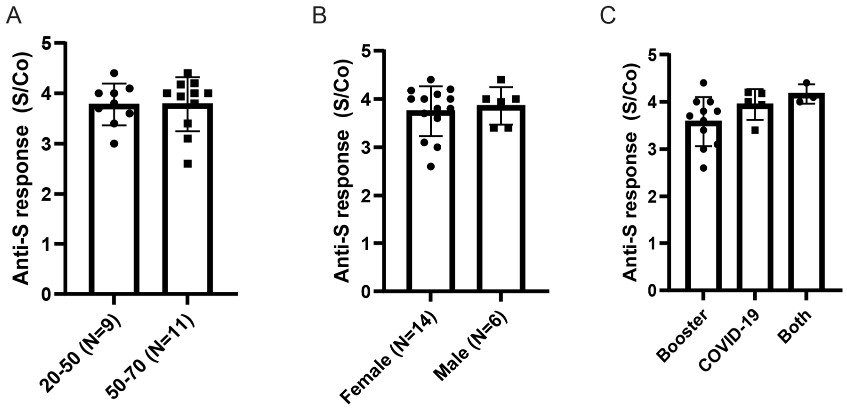

Supplementary Figure S2. Anti-Spike antibody response in HCW cohort at t3. (**A**) Comparison of anti-Spike antibody S/Co 14-16 months post vaccination in relation to participants age, (**B**) gender or (**C**) receipt of booster dose or self-reported COVID-19 infection or both. S/Co, Signal to cut-off ratio.

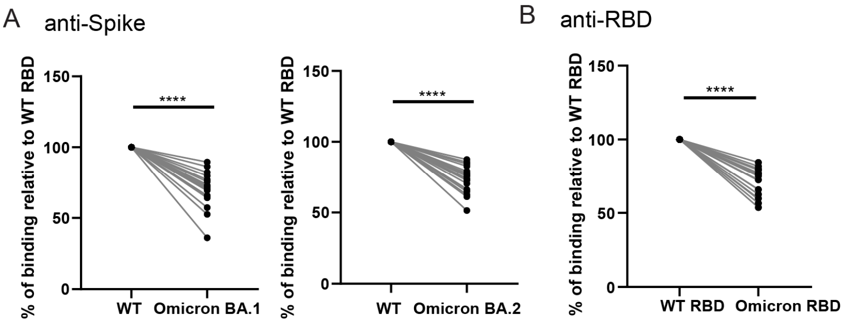

Supplementary Figure S3: Reactivity of 14-16 months (t3) vaccinees serum samples to Omicron variants. (**A**) Comparison of intensity of t3 serum binding to Omicron BA.1 and BA.2 Spike relative to binding to WT Spike protein. (**B**) Comparison of intensity of t3 serum binding to Omicron RBD protein relative to binding to WT RBD protein. Statistical test: paired t-test (A, B), **** p<0.0001.

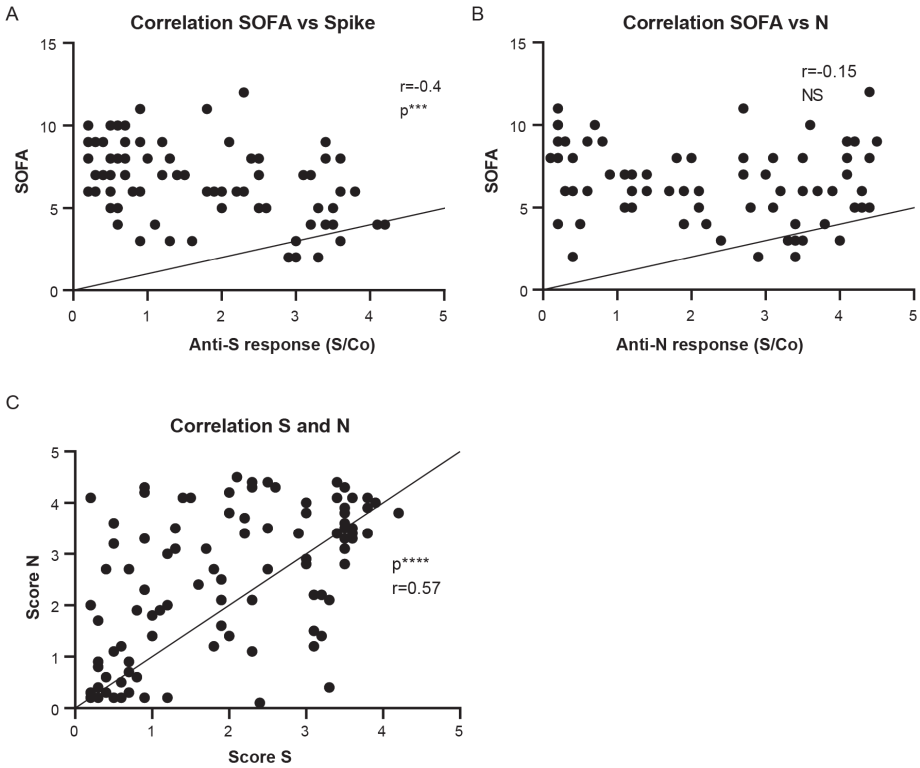

Supplementary Figure S4: Correlation analysis for hospitalized patients cohort**.** (**A**) Correlation between Spike antibody S/Co or (**B**) Nucleocapsid antibody S/Co to IVS patients SOFA score. (**C**) Correlation between anti-Spike and anti-Nucleocapsid antibody responses in samples from non-vaccinated COVID-19 patients (N=100). Statistical tests: Pearson correlation coefficients, p<0.0005.

Supplementary Table S1: Correlation analysis of anti-WT Spike S/Co vs. anti-Omicron S/Co for t2 HCWs.

	Omicron BA.1				*Omicron BA.2*				*Omicron RBD*			
Groups	**G6**	**G7**	**G8**	**G9**	**G6**	**G7**	**G8**	**G9**	**G6**	**G7**	**G8**	**G9**
WT	0.86 *	**0.94** ****	**0.96** **	0.86 ****	0.85 *	**0.93** ****	**0.94** **	0.87 ****				
WT RBD									0.85 ****	**0.93** *	**0.87** *	0.92 ****

*Numbers indicate r Pearson correlation coefficient, * indicate p-value significance.*
